# A case of IgA pemphigus with acantholysis in oral mucosal lesions

**DOI:** 10.1002/ski2.327

**Published:** 2024-01-06

**Authors:** Ayumi Yamanishi, Noriko Ono, Asako Tsunoda, Yasuhiko Asahina, Takayuki Fusumae, Jun Yamagami, Noriyasu Sakai, Norito Ishii, Masayuki Amagai, Hayato Takahashi

**Affiliations:** ^1^ Department of Dermatology Keio University School of Medicine Tokyo Japan; ^2^ Department of Dermatology Tokyo Medical University Tokyo Japan; ^3^ Department of Dermatology Kurume University School of Medicine Kurume Japan

## Abstract

IgA pemphigus is an autoimmune bullous disease caused by anti‐keratinocyte cell surface IgA autoantibodies. Mucous membrane involvement is rare in IgA pemphigus. We report a case of IgA pemphigus with oral mucosal lesions, in which acantholysis was pathologically confirmed. A 31‐year‐old woman presented with skin erythema with small pustules and oral mucosal erosions. Histopathological examination of the erosions on her oral mucosa and papules on her back revealed acantholysis and intraepidermal infiltration of neutrophils. Direct immunofluorescence tests showed intercellular deposition of IgA, but not IgG, mainly in the lower, but not entire, layer of the epidermis. C3 was linearly present in the basement membrane zone (BMZ), but not in the intercellular space. Enzyme‐linked immunosorbent assay revealed that both anti‐desmoglein (Dsg) 3 IgA and IgG were positive. Neither IgA nor IgG against desmocollin 1–3 were detected. This case was clinically and histologically compatible with IgA pemphigus, but immunologically anti‐BMZ autoimmunity was additionally observed. IgA pemphigus is classified into two major types: subcorneal pustular dermatosis type and intraepidermal neutrophilic dermatosis type. This case was not typical in terms of rarely observed oral lesions and predominant deposition of IgA in the lower layer of the epidermis. Instead, this case could be considered a rare subtype of IgA pemphigus, IgA‐pemphigus vulgaris. Oral lesions in IgA pemphigus may be clinical clue of having anti‐Dsg3 IgA that cannot be routinely examined.

## Graphical abstract

IgA pemphigus is a rare autoimmune bullous disease caused by anti‐keratinocyte cell surface IgA autoantibodies. Mucous membrane is usually not involved in clinical manifestation and acantholysis is not always observed in histopathology. In addition, evidence revealing pathogenic roles of anti‐desmoglein (Dsg) IgA in IgA pemphigus has been limited. We report the case of IgA pemphigus with oral mucosal lesions. This article not only demonstrates clear acantholysis but also implies the possible keratinocyte‐dissociating pathogenicity of IgA autoantibodies to Dsg3. Several interesting findings are condensed in this case.

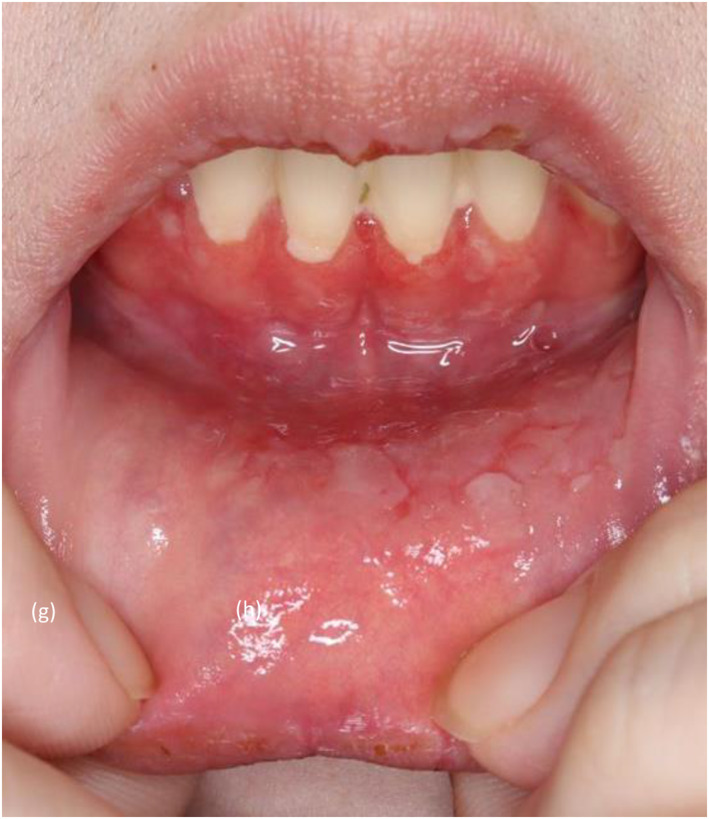

## 1 INTRODUCTION

IgA pemphigus is an autoimmune bullous disease caused by anti‐keratinocyte cell surface IgA autoantibodies.^1^ Clinically, flaccid pustules arise on an erythematous base and often appear in an annular arrangement, while mucous membrane involvement is rare.^2^ Herein, we report a case of IgA pemphigus with oral mucosal lesions showing acantholytic changes in histology and immunological features, including IgA reactivity to desmoglein (Dsg) 3 and C3 deposition in the basement membrane zone (BMZ).

## 2 REPORT

An otherwise healthy 31‐year‐old woman presented with skin erythema with small pustules and oral mucosal erosions. Her former doctor diagnosed IgA pemphigus based on the pathological and direct immunofluorescence (DIF) findings. Diamino‐diphenyl sulfone (DDS, 50 mg/day) was started but reduced (25 mg/day) due to methemoglobinemia and prednisolone (PSL, 20 mg/day) was added.

**FIGURE 1:**
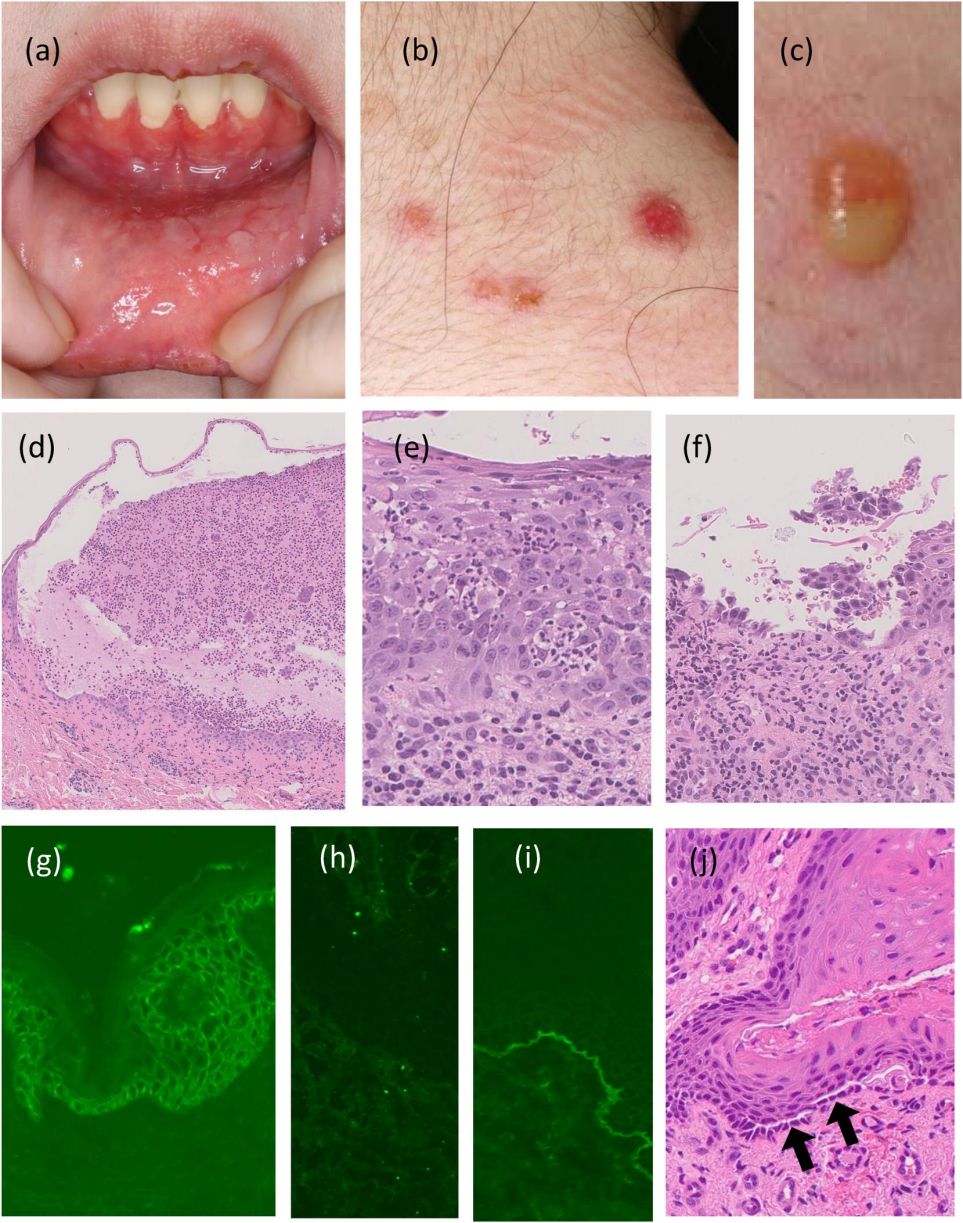
Clinical and histopathological features of the present case. (a) Multiple erosions of the oral mucosa. (b) Erosive erythema on the back. (c) Pustules on her chest. (d) Subcorneal neutrophilic infiltration observed in the pustule on the back (original magnification, ×100). (e, f) Erosions in the oral mucosa showing neutrophil infiltration in the entire epidermis (e) and acantholysis (f). (g–i) Direct immunofluorescence for IgA (g), IgG (h), and C3 (i). Intercellular deposition of IgA in the lower epidermis (g). No deposition of IgG (h). Linear C3 deposition at the BMZ (i). (j) Suprabasilar cleft (showed with black arrows) in the mucosal epithelium without neutrophilic infiltration in the normal‐appearing labial mucosa (original magnification, ×100). BMZ, basement membrane zone.

When she was transferred to our department, the symptoms were mild. DDS was discontinued and PSL was gradually reduced to 5 mg/day. Then, relapse occurred with erosions of the oral mucosa (Figure 1a), erosive erythema (Figure 1b), and pustules (Figure 1c) on the trunk. Upper gastrointestinal endoscopy revealed Nikolsky's phenomenon on the oesophageal mucosa.^3^

Histopathologically, pustules on her back revealed subcorneal neutrophil infiltration (Figure 1d), and erosions in the oral mucosa revealed acantholysis and intraepidermal infiltration of neutrophils (Figure 1e,f). DIF of the back skin and oral mucosa showed intercellular deposition of IgA (Figure 1g), but no IgG (Figure 1h). There was increased intensity of staining in the lower layer of the epidermis. C3 was linearly present in the BMZ, but not in the intercellular space (Figure 1i). Both indirect immunofluorescence (IIF) and salt‐split skin IIF were negative. Immunological findings are shown in Tables 1 and 2. Chemiluminescent enzyme immunoassay results were positive for anti‐Dsg3 IgG at 27 U/mL (positive ≥20), and enzyme‐linked immunosorbent assay (ELISA) revealed that both anti‐Dsg3 IgA and IgG were positive, 4 over (optical density, positive >0.15)^4^ and 83.48 (index value, positive ≥20), respectively. Neither IgA nor IgG against desmocollin 1–3 (Dsc1–3) were detected by ELISA. IgG against BP180, B230, and type VII collagen were also not detected by ELISA. The patient was treated with DDS (25 mg/day) and PSL (1 mg/kg/day, gradually tapered), and the lesions completely disappeared after 3 weeks without methemoglobinemia. Currently on 50 mg/day of DDS with PSL tapered to 6 mg/day, we have not seen any relapse of symptoms.

**TABLE 1:** Summary of histopathological findings and DIF results.

Site of lesion/cutaneous symptoms		Trunk/pustules	Oral mucosa/erosions	Labial mucosa/normal appearing
Histopathological findings	Acantholysis	+	+	+ (suprabasilar cleft)
	Neutrophilic infiltration	+	+	−
DIF	IgA	+	+	+
		Squamous intracellular: Increased intensity in the lower layer	Squamous intracellular: Increased intensity in the lower layer	Squamous intracellular: Increased intensity in the lower layer
	C3	+	+	+
		BMZ linear	BMZ linear	BMZ linear
	IgG	−	−	−

Abbreviations: BMZ, basement membrane zone; DIF, direct immunofluorescence.

**TABLE 2:** Summary of autoantibody analysis.

ELISA	ELISA	CLEIA
IgA (cut‐off value, unit)	IgG (cut‐off values)	IgG (cut‐off value, unit)
Dsg3 4 over (>0.15, OD)	Dsg3 83.48 (≥20, IV)	Dsg3 27 (≥20, U/mL)
Dsg1 0.005 (>0.15, OD)	Dsg1 0.56 (≥20, IV)	Dsg1 <3.0 (≥20, U/mL)
Dsc1 0.008 (>0.123, OD)	Dsc1 0.075 (>0.200, OD)	BP180NC16a <3.0 (≥9, U/mL)
Dcs2 0.004 (>0.048, OD)	Dcs2 0.080 (>0.070, OD)	
Dsc3 0.005 (>0.074, OD)	Dsc3 0.091 (>0.120, OD)	
	BP180NC16a 0.0 (≥9, IV)	
	BP230 1.7 (≥9, IV)	
	COL7 1.4 (mean 0.9, SD 1.04, U/mL)	

Abbreviations: CLEIA, chemiluminescent enzyme immunoassay; COL7, type VII collagen; Dsc, desmocollin; Dsg, desmoglein; ELISA, enzyme‐linked immunosorbent assay; IV, index value; OD, optical density.

## 3 DISCUSSION

IgA pemphigus is classified into two major types: subcorneal pustular dermatosis (SPD) and intraepidermal neutrophilic dermatosis (IEN). The autoantigen of SPD type was identified as Dsc1, while the antigen of IEN type remains unknown.^1^ This case may have elements of both SPD and IEN types because the pustules on her back showed subcorneal neutrophil infiltration (Figure 1d), while the erosive erythema on her oral mucosa showed neutrophil infiltration in the entire epidermis (Figure 1e). However, this case differed in IgA deposition pattern from typical SPD‐type cases in which IgA was observed in the upper epidermis, and from the IEN‐type cases in which IgA was observed in the entire epidermis, since IgA was predominantly deposited in the lower epidermis in this case.

In addition to these two types, the terms IgA‐pemphigus foliaceus (PF) and IgA‐pemphigus vulgaris (PV) were used for subtypes with IgA to Dsg1 and Dsg3, respectively in a review article.^5^ In this article, of the 49 cases of IgA pemphigus, 17 were SPD‐type, 12 were IEN‐type, 6 (12%) were IgA‐PV, 4 were IgA‐PF, 2 were IgA‐pemphigus vegetans, and 8 (16%) were not classified as any type of IgA pemphigus. They classified IgA pemphigus cases from clinical, histopathological, and immunological perspectives.^5^ This case may belong to IgA‐PV. In our case, although the ELISA was positive for both IgA and IgG against Dsg3, intercellular deposition of IgG was not detected in DIF and IIF. Therefore, the anti‐Dsg3 IgA autoantibodies may be pathogenic. Since the results of DIF and IIF demonstrated that circulating anti‐Dsg3 IgG was not able to bind to keratinocyte cell surfaces, IgG autoantibodies were not involved in the pathogenesis of this case.

A systematic review found oral mucosal involvement in 16 (13.2%) of 121 patients with IgA pemphigus.^1^ Among the 16 patients, 8 had anti‐Dsg3 IgA but no IgG reactivity.^1^ Another article reported oral mucosal symptoms were observed in 2 (33.3%) of six IgA‐PV patients.^5^ Since Dsg3 is highly expressed as a dominant adhesive molecule of desmosome in the oral mucosa,^6^ it would be reasonable that oral mucosal lesions are often seen in IgA‐PV than other types of IgA pemphigus. In our case, anti‐BMZ autoreactivity was also observed with C3 deposition in the BMZ. C3 deposition in the BMZ was reported in 3 (6.1%) of 49 cases of IgA pemphigus.^5^ Although anti‐BMZ autoreactivity usually causes oral lesions in the mucous membrane pemphigoid, subepithelial blister formation was histologically not observed in our case. Therefore, the pathogenic contribution of anti‐BMZ autoreactivity to the development of oral lesions in this case was unclear.

Histopathologically, acantholysis was not necessarily observed in IgA pemphigus and was reported in 17 (46.0%) of 37 cases.^5^ Only two previous reports^7^, ^8^ could provide pathological images confirming neutrophil accumulation and clear acantholysis, although these findings have been described in other reports. In our case, histopathology of the cutaneous and oral lesions showed acantholysis with neutrophilic infiltration (Figure 1f). In the histopathology of normal‐appearing labial mucosa, an intraepidermal cleft without neutrophil infiltration was unexpectedly observed at the suprabasilar level (Figure 1j). The pathomechanisms of acantholysis in IgA pemphigus remain unclear. Since neutrophil infiltration is always accompanied by acantholysis of IgA pemphigus, both the pathogenic contribution of IgA deposition and neutrophil infiltration to acantholysis need to be considered and cannot be separated. Histopathological findings in this case, suprabasilar cleft without neutrophil infiltration in the normal‐appearing labial mucosa possibly inferred pathogenic roles of anti‐Dsg3 IgA directly causing acantholysis, although experimental investigation is necessary to prove it. However, since functional disturbance of Dsg3 does not usually induce blister formation in the skin because of the compensatory roles of Dsg1 as known in classical pemphigus pathogenesis, pathogenic involvement of neutrophils in cutaneous blister formation despite the absence of anti‐Dsg1 autoreactivity was clinically implied in our case.

We presented a case of IgA pemphigus with oral involvement. Histopathology of the oral mucosal lesions showed acantholysis, and serologically, anti‐Dsg3 IgA autoantibodies were identified. Histopathological and immunological findings were characteristic and useful to consider pathogenic roles of IgA, C3, and neutrophils in this case. IgA pemphigus has been classified into two major types: SPD type and IEN type, but there are actually quite a few types that do not fit into these types. As exemplified by PF and PV, clinical symptoms differ depending on the target antigen of the autoantibody. Oral lesions in IgA pemphigus may be clinical clue of having anti‐Dsg3 IgA in the patients, extremely rare autoantibodies that cannot be routinely examined. Further accumulation of detailed clinical information is necessary to deepen our understanding of this disease features.

## CONFLICT OF INTEREST STATEMENT

The authors declare no conflicts of interest.

## AUTHOR CONTRIBUTIONS

**Ayumi Yamanishi**: Conceptualization (equal); formal analysis (equal); investigation (equal); methodology (equal); software (equal); visualization (equal); writing – original draft (equal). **Noriko Ono**: Conceptualization (equal); formal analysis (equal); investigation (equal); methodology (equal); project administration (equal); software (equal); supervision (equal); validation (equal); visualization (equal); writing – original draft (equal); writing – review & editing (equal). **Asako Tsunoda**: Writing – review & editing (equal). **Yasuhiko Asahina**: Writing – review & editing (equal). **Takayuki Fusumae**: Writing – review & editing (equal). **Jun Yamagami**: Writing – review & editing (equal). **Noriyasu Sakai**: Writing – review & editing (equal). **Norito Ishii**: Investigation (equal); methodology (equal); validation (equal); visualization (equal); writing – review & editing (equal). **Masayuki Amagai**: Supervision (equal); writing – review & editing (equal). **Hayato Takahashi**: Conceptualization (equal); funding acquisition (equal); investigation (equal); methodology (equal); project administration (equal); supervision (equal); writing – original draft (equal); writing – review & editing (equal).

## ETHICS STATEMENT

This study was approved by the institutional ethics committee of Keio University School of Medicine (approval number 20120180).

## Data Availability

Data sharing not applicable to this article as no datasets were generated or analyzed during the current study.
